# Immunofluorescence analysis reveals no increased seroprevalence of anti-*Bartonella schoenbuchensis*-IgG antibodies in German forest workers

**DOI:** 10.1186/s13071-025-06856-2

**Published:** 2025-08-04

**Authors:** Kim Nina Buntrock, Wibke Ballhorn, Heike Podlich, Johan Malmström, Lotta Happonen, Sounak Chowdhury, Annette Jurke, Volkhard A. J. Kempf

**Affiliations:** 1 Institute for Medical Microbiology and Infection Control, University Hospital, National Reference Laboratory for Bartonella Infections, Goethe University, Paul-Ehrlich-Str. 40, 60596 Frankfurt Am Main, Germany; 2 Division of Infection Medicine, Lund University, Lund, Sweden; 3 Department Infectious Disease Epidemiology, Northrhine Westphalia Centre for Health, Bochum, Germany; 4 Institute of Hygiene, University Hospital Muenster, University of Muenster, Muenster, Germany

**Keywords:** Deer keds, Zoonosis, Serodiagnostics, Exposition, Infection risk

## Abstract

**Background:**

*Bartonella schoenbuchensis *is suspected to cause deer ked dermatitis and febrile diseases in humans. Deer keds (*Lipoptena cervi*), which infest cervids (e.g., roe deer, fallow deer), are discussed as potential vectors for *B. schoenbuchensis*.

**Methods:**

We analyzed the seroprevalence of anti-*B. schoenbuchensis* immunoglobulin G (IgG) antibodies in sera of forest workers (FW; *n* = 82) compared to control sera of non-forest workers (NFW; *n* = 118) from North Rhine-Westphalia, Germany. For this purpose, an immunofluorescence assay (IFA) using Vero E6 cells infected with *B. schoenbuchensis* was established, and serum titers were assessed. Whole cell lysate of *B. schoenbuchensis* was introduced for analysis of seroreactivity by western blotting. Immunodominant proteins were identified by liquid chromatography–tandem mass spectrometry.

**Results:**

When using human sera, 54.9% (*n* = 45/82) of FW were tested positive at a titre ≥ 320 whereas IFA reactivity was 66.1% (*n* = 78/118) in NFW. When the cut-off titre was set to ≥ 640, then 18,3% (*n* = 15/82) of FW and 20,3% (*n *= 24/118) of NFW displayed seroreactivity, respectively. In immunoblot analysis, IFA-positive sera reacted with 18 different bands ranging from ca. 40–300 kDa. No elevated reactivity of sera from FW compared to those of NFW was observed.

**Conclusions:**

Our data speak against an increased seroprevalence of anti-*B. schoenbuchensis* IgG titers in FW, which are regularly exposed to deer keds, weakening the hypothesis that *B. schoenbuchensis* is transmitted to humans by deer keds.

**Graphical Abstract:**

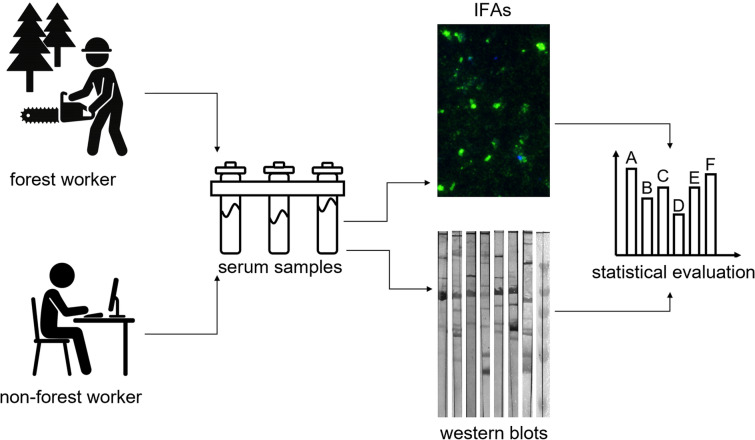

**Supplementary Information:**

The online version contains supplementary material available at 10.1186/s13071-025-06856-2.

## Background

Blood-sucking arthropods serve as vectors for numerous infectious agents in humans and animals, making them a significant focus of *One Health* approaches. Deer keds (*Lipoptena cervi*), members of the louse fly family (Hippoboscidae), are distributed across Europe, North America, and Siberia [1]. The deer ked (*L. cervi*) is recognized as a confirmed vector for *Bartonella schoenbuchensis,* a flagellated Gram-negative bacterium of the genus *Bartonella* causing “deer ked dermatitis.” The natural reservoir of *B. schoenbuchensis* predominantly comprises cervids, and the bacterium was first isolated from the blood of wild roe deer in 1999 [2].

The vector competence of *L. cervi* for *B. schoenbuchensis* appears to be well-established [3–9]. Occasionally, deer keds also bite humans, potentially causing dermatitis [10]. However, the transmission of pathogenic bacteria from the digestive tract of deer keds to humans during blood feeding has not been documented to date. Similar to the transmission mechanisms of other *Bartonella* spp. (*B. bacilliformis;* vector: *Lutzomyia verrucarum*; *B. quintana*: vector: *Pediculus humanus corporis*), there may be a hypothetical risk of *B. schoenbuchensis* being transmitted from *L. cervi* to humans. However, only one case of human infection presenting with chronic, non-specific symptoms has been reported [11], demonstrating at least that human infections with *B. schoenbuchensis* are not a frequent entity.

In popular media, the risk of *B. schoenbuchensis* infections transmitted by *L. cervi* is often exaggerated. In particular, the “yellow press,” alternative healthcare practitioners, and participants in animal and outdoor forums have issued warnings about *L. cervi* transmitting *B. schoenbuchensis* infections, claiming that they can cause “fatal heart inflammation" [12–18]. These assertions have generated unnecessary fear and uncertainty among patients despite lacking any evidence-based scientific foundation.

To investigate the actual human pathogenic potential of *B. schoenbuchensis*, this study aimed to compare systematically the prevalence of anti-*B. schoenbuchensis* immunoglobulin G (IgG) antibodies in human sera from a potential risk group (forest workers, FW) with those from a potential control group (non-forest workers, NFW). In this study we found no evidence of a specific increased seroreactivity in FW, arguing against a significant transmission risk of *B. schoenbuchensis* to humans.

## Methods

### Bacteria

*Bartonella schoenbuchensis* E251 R1 (DSM 13525) was grown for 4 days on Columbia agar plates (CBA; Becton Dickinson, Heidelberg, Germany) at 37 °C and 5% CO_2._ For the generation of defined bacterial stocks, agar plates were harvested, resuspended in lysogeny broth (LB) containing 20% glycerol, and stored at −80 °C. The number of viable bacteria per aliquot was determined by counting the colony-forming units (CFU) in serial dilutions from the frozen stocks after 14 days of cultivation. For some control experiments, *B. henselae* Marseille (CIP 104756) was used [19].

### Generation of a rabbit-anti-*B. schoenbuchensis* serum

*Bartonella schoenbuchensis* was grown on CBA plates for 4 days and harvested in sterile Dulbecco’s phosphate-buffered solution (DPBS, Gibco, Thermo Scientific, Rockford, IL, USA). Bacteria were inactivated with erythromycin (500 µg/ml DPBS) at 37 °C for 6 h and used as antigen for the production of anti-*B. schoenbuchensis* rabbit serum. For this, two rabbits were immunized on days 0, 7, 10, and 18, respectively, each with 5 × 10^7^ inactivated bacteria (Kaneka Eurogentec S.A., Seraing, Belgium), and serum was taken on day 28. The reactivity of the *B. schoenbuchensis* positive rabbit serum (including the pre-immune sera) was tested via immunofluorescence and western blotting. For this, *B. schoenbuchensis* was grown in *Bartonella* liquid medium [20] for 3 days, and bacteria were collected by centrifugation (4.991×*g*, 10 min) and washed three times in DPBS, and serum reactivity was assessed (data not shown).

### Human sera

All human sera were obtained from FW and office employees (= non-forest workers, NFW) in North Rhine-Westphalia. The FW cohort includes occupational groups (foresters, district managers, forest workers) with an increased occupational exposure to *L. cervi*, the presumptive vector of *B. schoenbuchensis*. The collection of the sera used in this study took place as part of the Study on Employees of Forestry Enterprises in North Rhine-Westphalia on the Epidemiology of Zoonoses led by Dr. Annette Jurke at the State Institute for Health of North Rhine-Westphalia. Details of this study have been published earlier [21]. For the study described herein, 200 sera (of which at least 200 µl serum was available) were randomly selected from the original study serum pool (*n* [FW] = 82; *n* [NFW] = 118).

### Generation of cell culture-derived *B. schoenbuchensis* antigen

For testing of human sera, a cell culture-derived *B. schoenbuchensis* antigen was systematically established according to the recommendations of the Centers for Disease Control and Prevention (CDC) [22]. Vero E6 cells (ATCC CCL-81) and HeLa-229 cells (DSMZ ACC-57) were cultured in RPMI-1640 medium (PAN Biotech, Aidenbach, Germany) supplemented with 10% heat-inactivated (30 min, 56 °C) fetal calf serum (FCS, Sigma-Aldrich, Taufkirchen, Germany), 1% non-essential amino acids and 1% sodium pyruvate (Merck Millipore, Darmstadt, Germany). Cells were detached by using trypsin–EDTA (Life Technologies, Thermo Fisher, Paisley, UK) for 5 min at 37 °C. For infection, 1.0 × 10^5^ Vero E6 or HeLa-229 cells were seeded in 24-well plates onto collagen-coated coverslips (collagen-G: 0.01% dissolved in DPBS; Matrix Bioscience, Mörlenbach, Germany) as described [19]. Infections were done in RPMI-1640 medium containing 10% FCS at a multiplicity of infection (MOI) of 100 with a centrifugation step (300×*g*, 5 min) to improve bacteria–cell contact. Infected cell cultures were incubated for 24 h, 48 h, and 72 h, respectively. Coverslips were washed three times (two times with pre-warmed RPMI-1640 medium without FCS and finally with DPBS), and cells were subsequently fixed with 3.75% paraformaldehyde (PFA) and 2% methanol (MeOH) dissolved in DPBS (pH 7.4). Antigen quality was assessed via immunofluorescence.

To produce cell culture antigen at a larger scale, Vero E6 cells were infected at a sub-confluent state in 80 cm^2^ cell culture flasks (Thermo Fisher, Roskilde, Denmark). Infection was performed as described above, and antigen was collected after 24 h, 48 h, and 72 h, each. For this, cell culture medium was removed and infected cells were washed three times (two times with pre-warmed RPMI-1640 medium without FCS and finally with DPBS) and detached by mechanical scraping with a rubber policeman. The resulting suspension was transferred to a 50 ml tube and further homogenized by firmly pressing (10 times) through a 20-gauge syringe needle. The resulting antigen was aliquoted and stored at −20 °C before use. *B. henselae* Marseille (CIP 104756) antigen was produced in parallel to be used as technical control (data not shown).

### Immunostaining of infected Vero E6 and HeLa-229 cells on coverslips

Coverslips with *B. schoenbuchensis*-infected Vero E6 and HeLa-229 cells were stained as previously described [19]. First, cells were permeabilized with 0.2% Triton X 100 (AppliChem, Darmstadt, Germany) for 15 min and unspecific reactions were blocked using 0.2% bovine serum albumin (BSA, Sigma) dissolved in DPBS (15 min, room temperature). Cells were incubated for 1 h using a rabbit anti-*B. schoenbuchensis*-serum (diluted at 1:6,400), and, subsequently for 1 h, using secondary IgG-antibodies (Alexa 488 conjugated goat anti-rabbit IgG, Jackson Immunoresearch, West Grove, PA, USA) Dako Agilent Technologies, CA, USA). The actin cytoskeleton was stained with Alexa 555 phalloidin (Invitrogen, Darmstadt, Germany) for 1 h together with the secondary antibodies. Nuclei and bacterial DNA were stained with 4′,6-diamidino-2-phenylindole (DAPI), 2 µg/ml; Merck, Darmstadt, Germany) for 10 min at 4 °C. All incubation steps were carried out in a humidified chamber and followed by three washes [twice with 0.2% Tween 20 (Carl Roth, Karlsruhe, Germany) in DPBS and finally in DPBS, each]. Slides were mounted using fluorescence mounting medium (Dako, Hamburg, Germany) and analyzed using a Zeiss Axio Imager 2 microscope equipped with a Spot RT3 camera (Diagnostic Instruments Inc., MI, USA) operated via the VisiView V.2.0.5 software (Visitron Systems, Puchheim, Germany). Antigen preparation was technically controlled using the *B. henselae* antigen and anti-*B. henselae* antibodies as described recently [19].

### Immunofluorescence analysis of human sera

The reactivity of patient sera with *B. schoenbuchensis* was evaluated using an immunofluorescence assay (IFA) with Vero E6 cell culture-derived antigen (MOI: 100). Antigen (40 µl; diluted 1:10 in DPBS) was applied to 12-well glass slides (StarFrost 12-well, Knittel Glas, Braunschweig, Germany), air-dried, and subsequently fixed and permeabilized for 15 min using 3.75% PFA and 2% MeOH (diluted in DPBS). To minimize nonspecific serum reactivity, the antigen was blocked with 0.2% bovine serum albumin dissolved in DPBS (4 °C, 12 h).

Human sera were serially diluted in DPBS (1:80, 1:160, 1:320, 1:640, 1:1,280, 1:2.560). Rabbit anti-*B. schoenbuchensis* serum (technical control) was diluted at 1:6,400, and the incubation was performed for 1 h at room temperature. Secondary antibodies (Alexa 488-conjugated donkey anti-human IgG (H + L) F(ab′)2-fragment IgG, 1:400; Jackson ImmunoResearch) were incubated for 1 h at room temperature. Negative controls (omitting human or rabbit sera) were included in all stainings (data not shown). The threshold titer of 320 was defined based on cut-off values established for other cell culture-based antigenic assays detecting *Bartonella* IgG antibodies [22].

Cell nuclei and bacterial DNA were stained with DAPI (2 µg/ml) for 10 min at 4 °C. All incubation steps were carried out in a humidified chamber and followed by three washes [twice with 0.2% Tween 20 in DPBS and finally in DPBS, each]. Slides were mounted using fluorescence mounting medium (Dako) and analyzed using a Zeiss Axio Imager 2 microscope (see above).

For control reasons, *B. henselae*-antigen was processed in parallel. For this, two human sera from patients with a *B. henselae* infection (originating from the serum biobank of the institute) were included. These sera showed reactivity at dilutions of 1:2.560 and 1:10.240, respectively, using a commercially available *B. henselae* immunofluorescence assay (Euroimmun, Lübeck, Germany) performed under strict quality-controlled criteria (laboratory accreditation according to ISO 15189:2014 standards; certificate number D–ML–13102–01–00).

### Preparation of *B. schoenbuchensis* antigen for SDS–PAGE and immunoblotting

*Bartonella schoenbuchensis* was cultured in *Bartonella* liquid medium [20] for 3 days at 37 °C with 5% CO^2^. Bacterial cells were harvested by centrifugation (4,991 × *g*, 10 min, 4 °C) and washed twice in DPBS. The resulting pellets were resuspended in SDS sample buffer (Laemmli buffer), heated at 70 °C for 10 min and further homogenized by firmly pressing (15 times) through a 27-gauge syringe needle. Sodium dodecyl sulfate–polyacrylamide gel electrophoresis (SDS-PAGE) was performed using 8% polyacrylamide gels as described previously [23].

For immunoblotting, proteins were transferred to nitrocellulose membranes (Amersham Protran 0.2 µm NC [nitrocellulose], Merck, Darmstadt, Germany). Membranes were cut into 3-mm strips and covered with human sera (diluted 1:100) in blocking buffer (Mikrogen Diagnostik, Neuried, Germany). Rabbit anti-*B. schoenbuchensis* IgG (see above) served as a technical control. After an incubation period of 1 h at room temperature under gentle shaking, membranes were washed three times with wash buffer (Mikrogen). Horseradish peroxidase (HRP)-conjugated secondary antibodies (rabbit anti-human IgG, 1:800, swine anti-rabbit IgG, 1:2,000, both Dako, Agilent Technologies, Santa Clara, CA, USA) were used to detect anti-*B. schoenbuchensis* IgG. Blots were developed using 3,3′,5,5′-tetramethylbenzidine liquid substrate (TMB; Mikrogen) for ca. 5 min, and the reaction was stopped with three washes with distilled water. The strips were dried on paper towels.

### Immunoblot analysis of human sera

The seroreactivity of 200 human serum samples with *B. schoenbuchensis* was further assessed in western blots. For this, immunodominant bands were assessed for their reactivity. Using a protein marker (PageRuler Plus 250–10 kDa prestained; Thermo Fisher Scientific), the specific molecular weights of the particular immunodominant bands were determined. Additionally, the intensity of each band was scored with the following values: 0.1 points (very faint), 0.5 points (faint), 1 point (strong), or 2 points (very strong). The scores of all bands of each serum sample were summarized to the band reactivity sum (“band sum”) similar to established and commercially available serodiagnostic tests (e.g., Mikrogen *recom*Line Borrelia IgM and IgG®, Mikrogen) [24, 25].

### Sample preparation for mass spectrometry

In-gel digestion of the proteins in the immunodominant bands was done essentially as described [26]. Briefly, the gel bands were destained with 100 mM ammonium bicarbonate, followed by shrinking with 100% acetonitrile. The gel pieces were saturated with trypsin (Promega V5111, Promega, Madison, WI, USA) on ice for 2 h, and the reaction was incubated further overnight at 37 °C. The peptides were extracted with 5% formic acid in 100% acetonitrile (both Sigma-Aldrich) for 15 min at 37 °C, and the collected supernatant was dried using a SpeedVac (miVac DUO concentrator, GeneVac, SP Scientific, Gardiner, NY, USA). Dried peptides were reconstituted in 0.1% formic acid in 2% acetonitrile, followed by further sample purification using SP3 beads (Thermo Scientific) according to the manufacturer’s protocol. The purified peptides were reconstituted in 0.1% formic acid in 2% acetonitrile containing 1:10 iRT peptides (custom ordered at JPT Peptide Technologies GmbH, Berlin, Germany) [27].

### Liquid chromatography–tandem mass spectrometry (LC–MS/MS)

All proteomics data were collected in data-dependent acquisition (DDA) mode on a Q Exactive HF-X instrument (Thermo Scientific) connected to an Easy-nLC 1200 system (Thermo Scientific). The peptides were loaded and concentrated on an Acclaim PepMap 100 C18 precolumn (75 μm × 2 cm) and then separated on an Acclaim PepMap RSLC column (75 μm × 25 cm, nanoViper, C18, 2 μm, 100 Å) (both columns Thermo Fisher Scientific, Waltham, MA, USA), at a column temperature of 45 °C and a maximum pressure of 900 bar. A linear gradient of 4% to 45% of 80% acetonitrile in aqueous 0.1% formic acid was run for 60 min. In total, 1 full MS scan (resolution 60,000; mass range of 390–1210 m/z) was followed by MS/MS scans (resolution 15,000) of the 20 most abundant ion signals. The precursor ions were isolated with a 2.6 m/z isolation window and fragmented using higher-energy collisional-induced dissociation (HCD) at a normalized collision energy of 30. The dynamic exclusion was set to 45 s.

Acquired MS raw spectra were analyzed using Proteome Discoverer 2.5 (Thermo Fisher Scientific) against an in-house compiled dataset containing the *B. schoenbuchensis* proteome, including common contaminants. Fully tryptic digestion was used, allowing two missed cleavages. Carbamidomethylation (C) was set to static, and protein N-terminal acetylation and oxidation (M) to variable modifications. Mass tolerance for precursor ions was set to 10 ppm, and for fragment ions to 0.06 Da. The protein false discovery rate (FDR) was set to 1%. Proteins quantified by two or more unique peptides were considered relevant. For identification, the gel bands were filtered for high-confidence identification (proteins identified as “peak found” or “not found” per gel band were excluded) and sorted by highest abundance, respectively. The identified proteins are presented in Supplementary Table 1.

### Testing of human sera for cross-reactivity

A subset of human sera strongly reactive in *B. schoenbuchensis* IFA testing (positive with titers ≥ 640) was selected (n = 20) and tested for IgG cross-reactivity with the following commercially available diagnostic tests: *B. henselae, B. quintana* IFA (positive titer ≥ 320, each; Euroimmun, Lübeck, Germany), *Mycoplasma pneumoniae* enzyme-linked immunosorbent assay (ELISA)-(positive ratio > 1.1; Euroimmun), *Brucella* spp. ELISA (positive ≥ 30 U/ml; Virion/Serion, Würzburg, Germany), *Chlamydophila pneumoniae* microimmunofluorescence (positive titer ≥ 320), *Rickettsia* spp. IFA (positive titer ≥ 320; Focus Diagnostics, CA, USA), *Coxiella burnetii* IFA (positive titer ≥ 16; Fuller Laboratories, CA, USA), *Treponema pallidum* TPPA (Fujirebio Diagnostics, Tokyo, Japan), and *Yersinia* spp. ELISA (positive ≥ 24 U/ml; Mikrogen) according to the respective manufacturer’s instructions. All laboratory testing was conducted under strict quality-controlled conditions according to ISO 15189:2014 standards (certificate number D–ML–13102–01–00, valid through January 25, 2025), at the Institute for Medical Microbiology and Infection Control, University Hospital Frankfurt am Main, Germany.

### Statistical analysis

The Mann–Whitney U test was employed to detect differences in IgG reactivity between the experimental and control groups. Due to the sample size (n > 50), the U statistic was transformed into a z-score. For a confidence interval of 0.05, *z*-scores below –1.96 or above 1.96 were considered statistically significant. Subsequently, the difference between the two cohorts (FW vs. NFW) in the prevalence of anti-*B. schoenbuchensis* IgG antibodies was analyzed using the Mann–Whitney U test. To examine the relationship between the results obtained from the immunoblots and those from the indirect immunofluorescence assays, a Spearman rank correlation analysis was performed.

## Results

### Establishment of an immunofluorescence assay for detecting anti-*B. schoenbuchensis* IgG antibodies

To date, no validated protocol for serodiagnosis of human anti-*B. schoenbuchensis* antibodies has been published. To develop an immunofluorescence assay (IFA) for this purpose, a cell culture-antigen-based approach, previously described for *B. henselae* and *B. quintana* [22, 28], was adapted. For antigen preparation, Vero E6 cells [22] or HeLa-229 cells [29] were selected as underlying cell cultures. These cells were infected with *B. schoenbuchensis* (see Materials and Methods) and incubated for 24 h, 48 h, and 72 h, respectively. Following incubation, infected cells were stained with rabbit serum containing anti-*B. schoenbuchensis* IgG antibodies. These antibodies were produced before by immunizing rabbits with erythromycin-inactivated bacteria (see Materials and Methods).

**Fig. 1:**
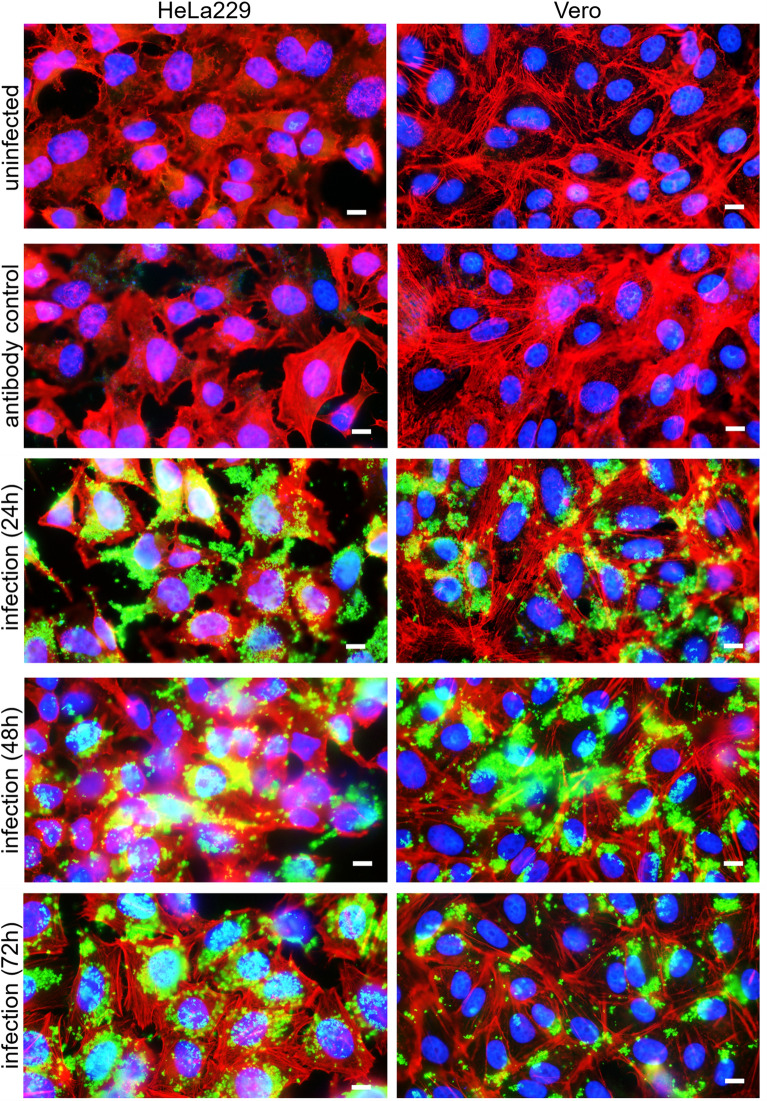
Development and quality control of *B. schoenbuchensis*-antigen used for immunofluorescence analysis. HeLa-229 cells (left) and Vero cells (right) were infected with *B. schoenbuchensis* (MOI 100) for 24, 48, and 72 h, respectively. Uninfected cells and cells stained only with the secondary antibody (Alexa 488 conjugated goat anti-rabbit IgG antibodies) served as staining controls. Red: actin (stained by TRITC-Alexa-555-conjugated phalloidin), blue: nuclei (stained by DAPI), green: *B. schoenbuchensis* (primary antibody: rabbit anti-*B. schoenbuchensis,* secondary antibody: Alexa 488 conjugated goat anti-rabbit IgG). Scale bar: 10 µm

Fixation protocols using 3.75% paraformaldehyde (PFA) with or without 2% methanol (MeOH, both prepared in DPBS) were compared, demonstrating slightly enhanced fluorescence signals with PFA/MeOH fixation (data not shown). At all three time points, fluorescence microscopy revealed densely infected cells. To minimize potential cross-reactivity with human-origin cells, subsequent analyses utilized antigens derived from Vero E6 cells (in accordance with existing protocols) [22, 28]. Antigen preparations from all three time points were pooled to ensure inclusion of (unknown) bacterial early- and late-stage infection proteins. All evaluation steps were technically controlled by conducting parallel *B. henselae*-IFA testing as earlier described [30] (data not shown). This process resulted in a robust antigen preparation suitable for further analyses of human sera (Fig. 1).

### Analysis of human sera from forest workers and non-forest workers for anti-*B. schoenbuchensis* IgG (IFA)

**Fig. 2:**
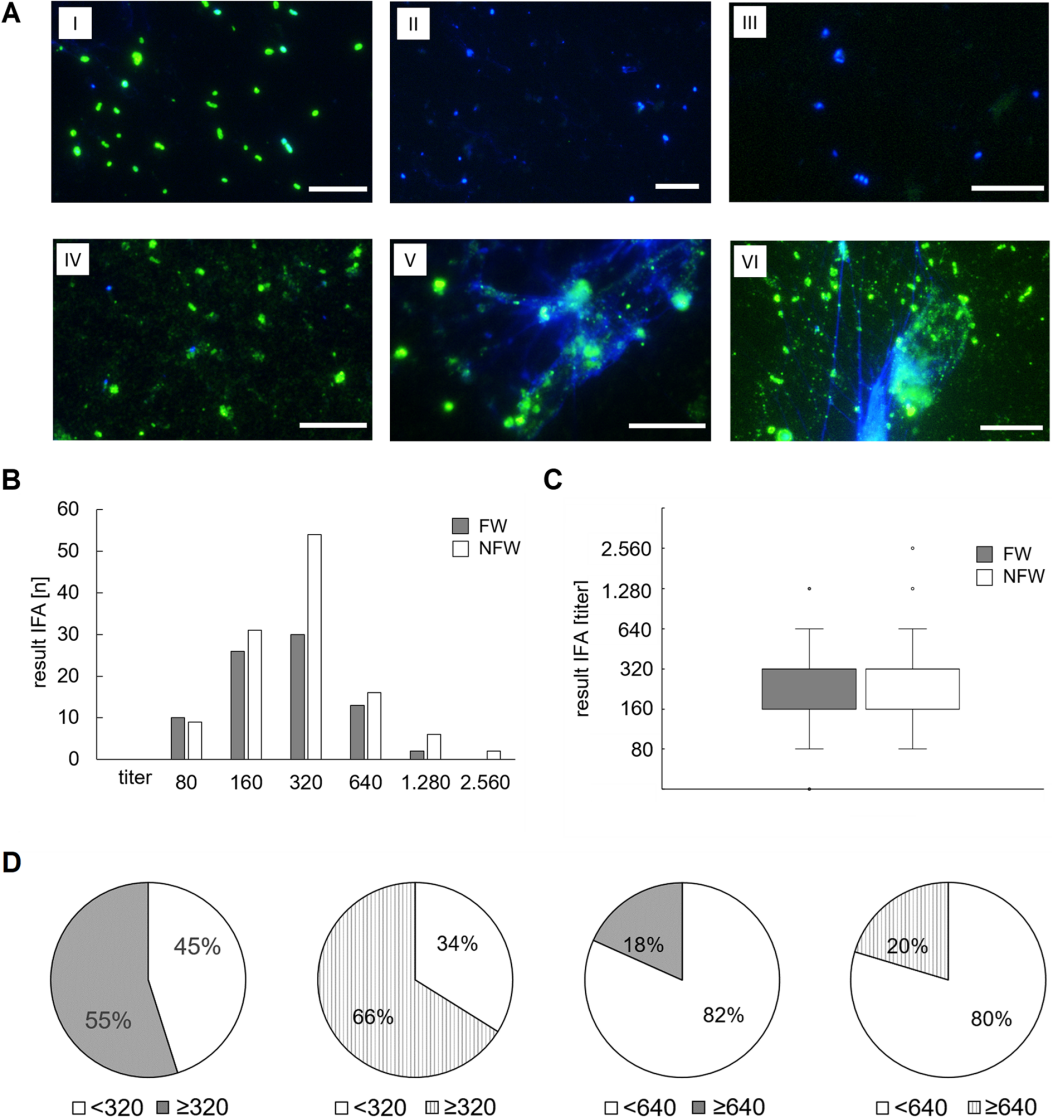
Immunofluorescence analysis of sera from forest workers (FW; *n* = 82) and non-forest workers (NFW; *n* = 118). **A** IFA staining of selected serum samples. (Vero cell antigen) I: positive control (rabbit anti-*B. schoenbuchensis*) II: negative control (anti-rabbit antibody), III: negative control (anti-human antibody), IV: serum NFW “NRW020” (titer: 80), V: serum NFW “NRW007” (titer: 320), VI: serum NFW “NRW119” (titer: 2.560). Green: bacteria, blue nuclei (DAPI). Scale bar: 10 µm. **B** Absolute frequencies of individual serum titers depicted for both FW and NFW, respectively. **C** Statistic analysis (box plot) of IFA titers of sera from FW and NFW. The median and the interquartile range are shown. **D** Pie chart of reacting sera at two cut-off titers (320, 640). 55% of the FW sera (*n* = 45) and 66% of the NFW sera (*n* = 78) were reactive at a cut-off titer of 320 whereas 18% of the FW sera (*n* = 15) and 20% of the NFW sera (*n* = 24) were reactive at a cut-off titer of 640

To optimize the detection protocol, a suitable secondary antibody was first selected. Two highly *B. henselae*-reactive human sera (titers: 1:2,560 and 1:10,240) were tested first with *B. henselae* antigen, resulting in the identification of an appropriate secondary antibody (see Materials and Methods; data not shown). Subsequently, 82 randomly selected sera from FW and 112 sera from NFW were analyzed by IFA (Fig. 2). Considering the unpredictability of results, strict technical controls were implemented, utilizing the described anti*-B. schoenbuchensis* rabbit serum. Serum titers ranged from < 80 to 2.560, with no clear differentiation between FW and NFW. Using a threshold titer of 320, seroprevalence was ~ 55% in the group of FW (*n* = 45) and ~ 66% (*n* = 78) in the group of NFW. With a threshold of 640, seroprevalence was ~ 18% (*n* = 15) for the FW and ~ 20% (*n* = 24) for the NFW. No statistically significant difference was observed between FW and NFW groups.

**Fig. 3:**
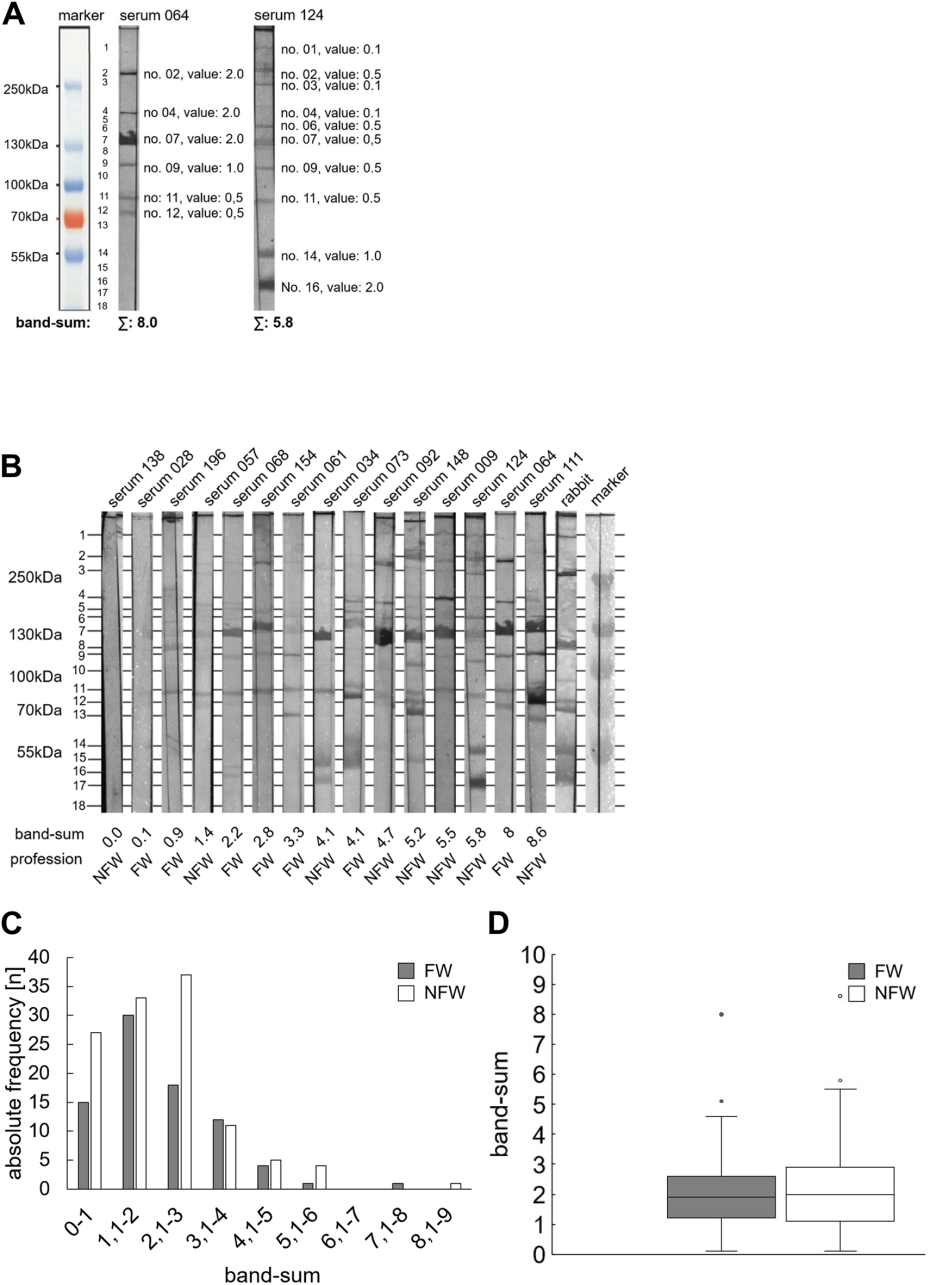
Western blot analysis of sera from forest workers (FW; *n* = 82) and non-forest workers (NFW; *n* = 118). **A** Development and exemplary demonstration of the band-sum calculation (serum 064 and 124, respectively). Every band was given a value (0.1, 0.5, 1.0, or 2.0) according to their relative intensity, and all band values were summed up to (serum 064: ∑ 8.0, serum 124: ∑ 5,8). **B** Representative western blot analysis of 15 sera from FW (*n* = 7) and NFW (*n* = 8) with their respective band sums. For positive control, a rabbit-anti *B. schoenbuchensis* serum was used. **C** Absolute frequency of the particular band sums of sera from FW and NFW. **D** Statistic analysis (box plots) of the band sums of sera from FW and NFW. The median and the interquartile range are shown

### Analysis of human sera from forest workers and non-forest workers for anti-*B. schoenbuchensis* IgG (western blot)

**Fig. 4:**
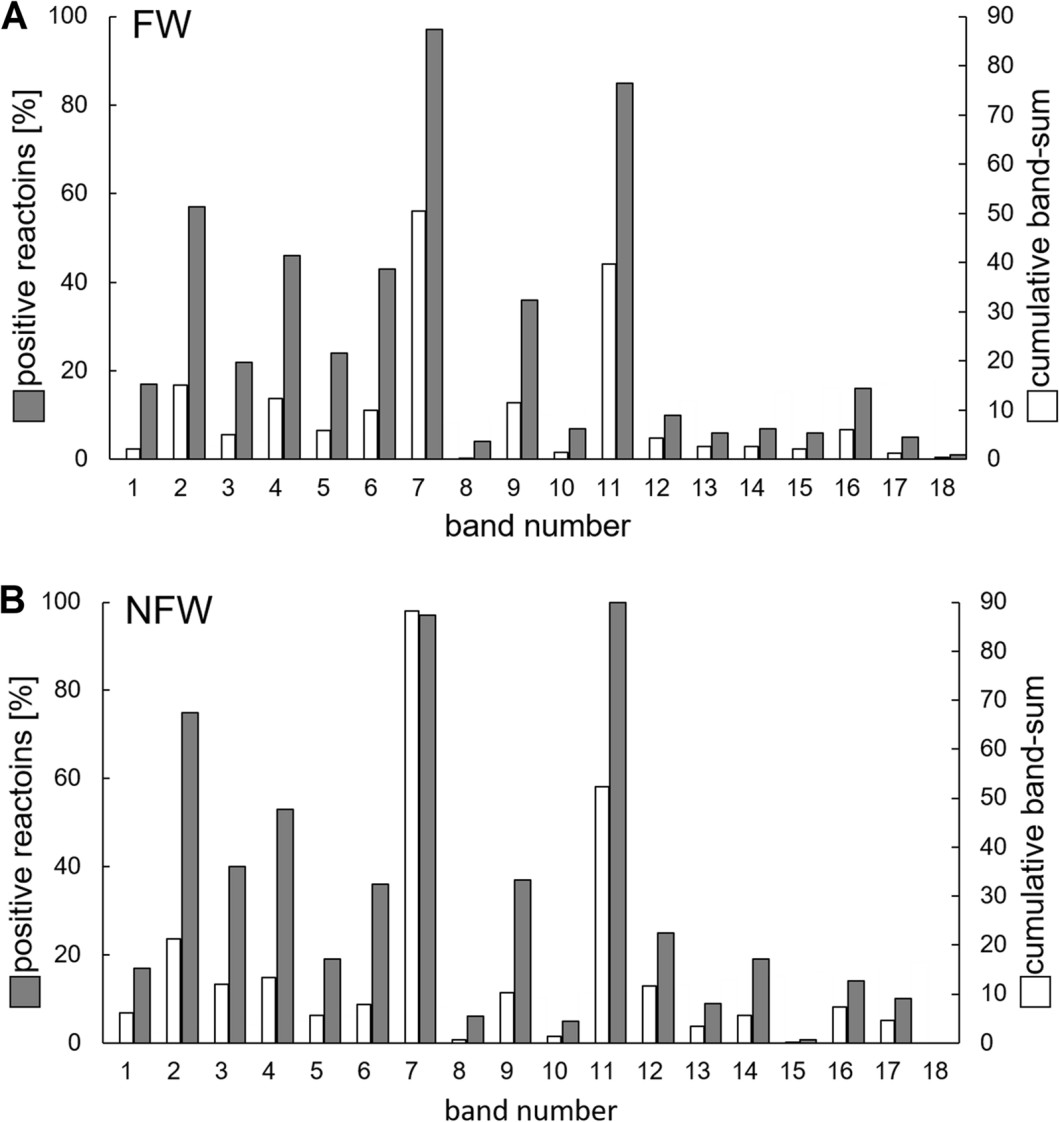
Band reactivity of sera in western blot analysis.** A** Forest workers (FW; *n* = 82),** B** non-forest workers (NFW; *n* = 118). The percentage of sera reacting with a specific band (independent from the band intensity) is given in grey, and the cumulative band sum of all sera is given in white

Given the lack of differentiation between FW and NFW in IFA results and the unexpectedly high seroreactivity, western blot analysis was performed. A protein preparation from liquid-grown *B. schoenbuchensis* was used to prepare western blots. Serum reactivity was assessed by scoring the intensity of reactive bands for each serum, following established diagnostic principles in infection serology (e.g., *Borrelia* antibody detection using the Mikrogen recomLine *Borrelia*® test, [25]. As a technical control, the rabbit anti-*B. schoenbuchensis* serum was included. Western blot analysis revealed a band reactivity range of 0.1 to 9, again showing no clear differentiation between FW and NFW (Figs. 3, 4) and corroborating the results from IFA testing.

### Identification of immunodominant bands by mass spectrometry

To identify potential serological markers for *B. schoenbuchensis* infection, immunodominant bands (6, 7, 9–16) detected in western blots were analyzed via LC–MS/MS. western blots were overlaid with Coomassie-stained gels, and matching bands were excised for MS-based identification. Collectively, *n* = 166 *B. schoenbuchensis* proteins were identified at high confidence across the 10 gel bands, out of which 28 were hypothetical, uncharacterized proteins (Supplementary Table 1). Most bands (6, 7, 9, 12, 14, 15, and 16 generated confident protein identifications (band 6: DNA-directed RNA polymerase subunit beta [accession FFM_00798]; band 7: hypothetical protein [FFM_01127]; band 9: hypothetical protein [FFM_00135]; band 12: chaperone protein DnaK [FFM_00274]; band 14: 60 kDa chaperonin [FFM_01167]; band 15: succinyl-diaminopimelate desuccinylase [FFM_00379]; band 16: ATP synthase subunit alpha [FFM_00120]), while bands 10, 11, and 13 generated inconclusive protein identifications (band 10: outer membrane protein assembly factor BamA [FFM_00816] and NAD-dependent malic enzyme [FFM_00853]; band 11: GTP-binding protein TypA/BipA [FFM_00420] and chaperone protein DnaK [FFM_00274], and band 13: transcription termination/anti-termination protein NusA [FFM_00349] and 30S ribosomal protein S1 [FFM_00299]). However, as no seroreactivity differences between FW and NFW were detected in western blots, further analyses of these data were not pursued.

### Determination of cross-reactivity of sera of forest workers and non-forest workers

**Fig. 5:**
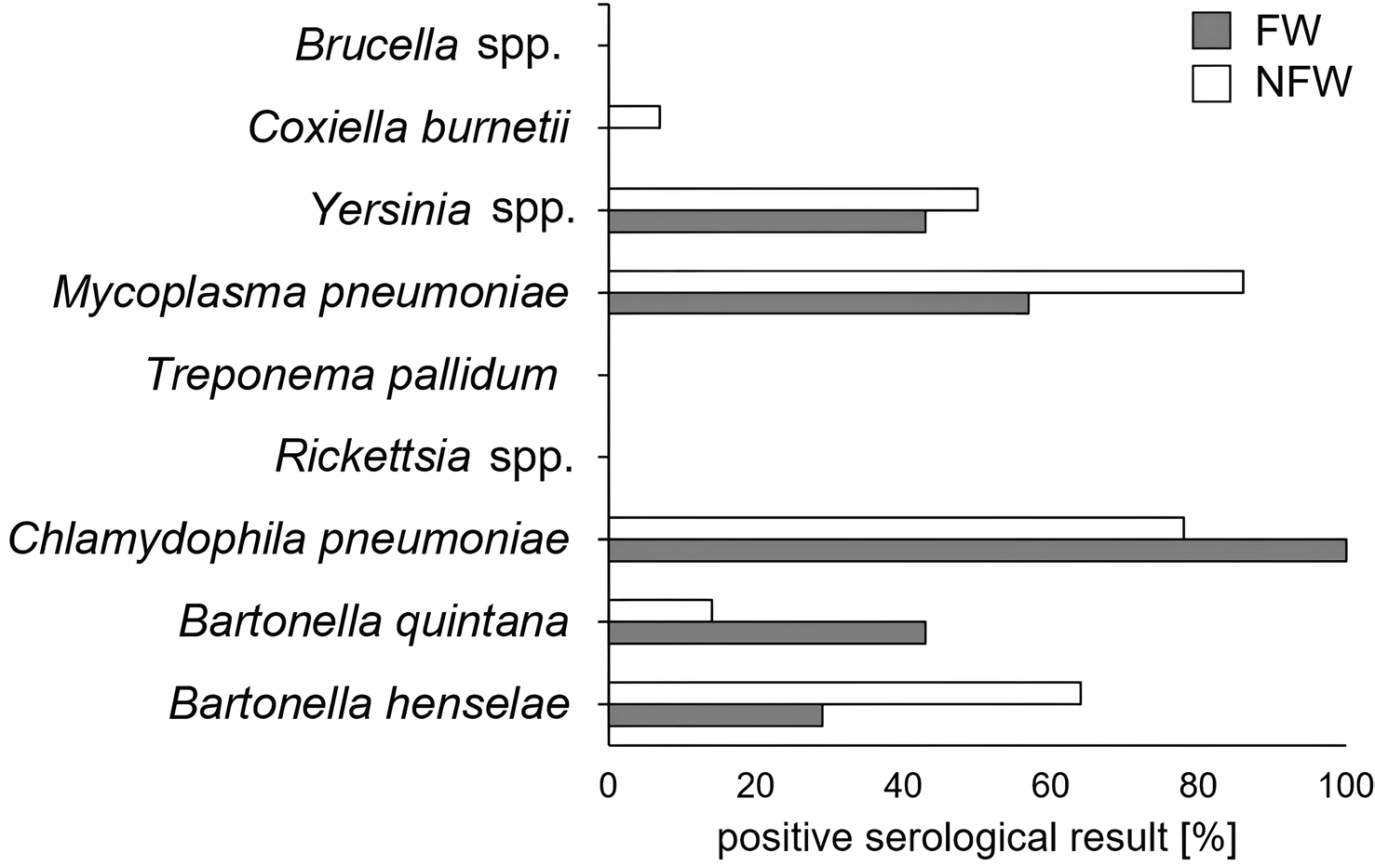
Cross-reactivity of sera from selected FW forest workers (FW; *n* = 7) and non-forest workers (NFW; *n* = 14). Sera were tested for cross-reactivity using commercially available serological tests under certified laboratory conditions. Cross-reactivity is given as the percentage of sera showing cross-reactivity in the specific serodiagnostic testing

Finally, the potential for nonspecific serological cross-reactivity was investigated to explain the high percentage of seroreactivity against *B. schoenbuchensis* in IFA and western blot testing in both FW and NFW groups. Selected sera (FW; *n* = 7; NFW; *n* = 14) reactive in IFA testing were further analyzed using commercial diagnostic assays for antibodies against *Brucella* spp., *Coxiella burnetii*, *Yersinia* spp., *Mycoplasma pneumoniae*, *Treponema pallidum*, *Rickettsia* spp., *Chlamydophila pneumoniae*, *B. quintana*, and *B. henselae*. Results indicated that the potential for cross-reaction is high, and every serum exhibited at least one cross-reactivity with the tested pathogens. The highest amount of cross-reactivity was observed for *M. pneumoniae* (FW: *n* = 4, 57%; NFW *n* = 12, 86%) and *C. pneumoniae* (FW: *n* = 7, 100%; NFW: *n* = 11, 78%). No cross-reactivity was detected for *Brucella* spp., *T. pallidum*, or *Rickettsia* spp. Sera show cross-reactivity for *C. burnetii* (FW: n = 0, 0%; NFW n = 1, 7%), *Yersinia* spp. (FW: *n* = 3, 43; % NFW: *n* = 7, 50%), *B. quintana* (FW: *n* = 3, 43%; NFW: *n* = 2, 14%) and *B. henselae* (FW: *n* = 2, 20%; NFW: *n* = 9, 64%) (Fig. 5).

## Discussion

The deer ked (*L. cervi*) is distributed in Europe, North America, and Siberia and mainly infests cervids (roe deer, fallow deer, moose). *Bartonella schoenbuchensis* bacteremia was demonstrated in ∼80% of the roe deer analyzed in Germany [2]. From a one health perspective, deer keds represent a potential vector for *B. schoenbuchensis* as detected in up to 72% of deer keds collected in Germany *B. schoenbuchensis* [31].

Many *Bartonella* spp., which are pathogenic for humans, are transmitted by various arthropods and are therefore anthropozoonotic agents [e.g., *B. quintana*: body lice (*P. humanus*), *B. henselae*: cat fleas (*Ctenocephalides felis*), *B. bacilliformis*: sand flies (*L. verrucarum*)]. Controversial evidence exists for the transmission of *Bartonella* spp. to humans by ticks. Although up to 40% of *Ixodes ricinus* ticks harbor, *e.g. B. henselae* DNA [32], this way of transmission is still not established [33]. Little is known about the zoonotic risk coming from *Bartonella* spp. infecting ruminant hosts of deer keds. Given the close association of the deer ked with its ruminant hosts, including regular blood meals, and the incidental infestation of humans with this arthropod, deer keds could theoretically serve as vectors for the transmission of *B. schoenbuchensis* to humans [3, 31]*.* Such an occasional transmission risk to humans is obviously apparent to, *e.g.* FW, hunters or cross-country runners [3].

However, because *B. schoenbuchensis* infections are often asymptomatic in their natural host, little is known about the health impacts for humans and animals. To date, it is unclear whether and which human diseases might be caused by *B. schoenbuchensis*, potentially transmitted by deer keds. Upon ked bites, the so-called deer ked dermatitis might occur, which is characterized by persistent, therapy-resistant, pruritic papules. These skin reactions can form 1 to 24 h after deer ked contact, and it was shown that immunologic mechanisms are probably involved in the pathogenesis [10]. It was proposed that *B. schoenbuchensis* might act as a cause of this dermatitis because of the similarity to the primary manifestation of cat scratch disease caused by *B. henselae* [3]. However, to the best of our knowledge, the direct contribution of *B. schoenbuchensis* in the development of deer ked dermatitis has never been demonstrated. In fact, the pathogenic potential of *B. schoenbuchensis* for humans is unclear, as any proven evidence for this assumption is missing. So far, the bacterium has only once been isolated from the blood of a patient suffering from fatigue, muscle pain, and fever. This patient had a history of tick bites and was seronegative for Lyme borreliosis [11]. Therefore, there is very limited evidence for a relevant human pathogenic potential of *B. schoenbuchensis* in general. In this context, it should at least be noted that both transstadial and transovarial transmission of *B. schoenbuchensis* in ticks may imply a potential, albeit theoretical, risk of *Ixodes ricinus* serving as a vector to humans [34].

We proposed earlier that the presence of anti-*B. schoenbuchensis* antibodies in deer ked-exposed patients would need to be systematically analyzed [31] to explain whether deer ked transmitted *B. schoenbuchensis* might contribute to the pathogenesis of deer ked dermatitis or other manifestations [31]. However, serological tools to perform such surveys are not commercially available, and moreover, no cut-off values for *B. schoenbuchensis* serology have ever been determined. In this first systematically conducted study, we compared the seroprevalence of anti-*B. schoenbuchensis* antibodies in a presumably highly deer-ked exposed cohort (FW) with that in a control cohort (NFW) by using pre-existing sera from a former study [21]. The establishment of a.

*Bartonella schoenbuchensis* serodiagnostics were performed as carefully as possible by using a CDC-guided IFA protocol or by performing western blots (both in accordance with *B. henselae*) [22, 30] with band intensity sum analysis [25] and extensive inclusion of numerous technical controls. The conclusion of our results is very clear: neither IFA nor western blot testing revealed elevated seroreactivity in FW compared to NFW clearly underlined by statistical analysis. On a more critical evaluation it must be mentioned that the percentage of reactive sera with 55–66% at a titer of 320 and even with 18–20% at a titer of 640 raises concerns regarding the specificity and the cross-reactivity of the detected antibodies from human sera (Fig. 2). An accurate assessment of the diagnostic utility of the assay could only be performed if a set of sera from patients with confirmed *B. schoenbuchensis* infection were available for analysis in our assay. Unfortunately, we are unaware of the existence of such a serum collection.

Although only sera with a titer of ≥ 640 were included, the unexpectedly high rate of cross-reactivity observed in this first seroepidemiological study of *B. schoenbuchensis* IgG antibodies in humans worldwide remains unexplained. In fact, it is known by serological analyses that ~ 10% of human sera reactive with *B. henselae* show cross reactivity with other bacteria, especially with *Mycoplasma* spp. [35] and *Chlamydia* spp. [36]. We also observed that every tested anti-*B. schoenbuchensis*-positive serum showed cross-reactivity with at least one of the potentially tested cross-reactive antigens (Fig. 5). The highest cross-reactivity of sera was seen for *M. pneumoniae* with 57–86% and with *Chlamydophila* spp. with 78–100% (Fig. 5). Therefore, we cannot definitively estimate the extent to which the serological results of our investigations might be distorted by cross-reactivities.

As a national reference center, we receive approximately 10–20 requests per year from concerned individuals who worry about contracting a dangerous infection with

*B. schoenbuchensis* following a deer ked bite. This is primarily due to the sensationalistic and alarmist reporting by some media outlets, which repeatedly and drastically warn of conditions such as “dangerous endocarditis” [12–18].

So far, there is no strong evidence supporting the potential of *B. schoenbuchensis* to infect humans. Based on our findings, as well as the existing literature, it remains unclear why the Central Committee for Biological Safety (ZKBS), Germany, classified *B. schoenbuchensis* as a “pathogen with zoonotic potential” [37] which might lead to a similar classification by other authorities in other countries. Our study, along with our novel literature analysis, found no conclusive evidence supporting any specific infection risk for humans posed by *B. schoenbuchensis*. Therefore, our results largely rule out the infectious potential of this pathogen in human medicine.

## Conclusions

Infestations of humans by deer keds are often met with strong emotional reactions, particularly among medical laypersons. However, whether *B. schoenbuchensis* can cause human infections at all remains a topic of debate, as no strong evidence supporting this has been documented. As a national reference center, we frequently receive inquiries from individuals and their doctors concerned about exposure to deer keds. Our analysis whether anti-*B. schoenbuchensis* antibodies are more frequently detected in FW (who are regularly exposed to deer keds) compared to NFW demonstrates no elevated seroprevalence in FW, indicating that they might not be at elevated risk for an exposition to this bacterium. These results therefore contribute to countering unsettling statements suggesting that deer keds might transmit *B. schoenbuchensis* infections to humans.

## Supplementary Information

Supplementary material 1: Supplementary Table 1: Identification of immunoreactive bands by LC–MS/MS. The table lists the confidence of the identification (protein false discovery rate [FDR] Confidence: Combined), the protein accession ID (Accession), the protein name (Description), the calculated peptide coverage (Coverage [%]), the number of peptides identified (# Peptides), the number of protein unique peptides identified (# Unique Peptides), the calculated protein length based on the sequence database (#AAs), the calculated molecular weight based on the sequence database (MW [kDA]), the relative protein abundance in each gel band (Abundance) and whether a protein has been identified in a given sample or not [high, peak found or not found].

## Data Availability

Data supporting the main conclusions of this study are included in the manuscript.

## Declarations

### Statement

Some parts of the text were improved in clarity and style during manuscript preparation with the assistance of ChatGPT (OpenAI, San Francisco, CA). The authors reviewed and edited all content for accuracy and originality.
