# Correction: Immunofluorescence analysis reveals no increased seroprevalence of anti-*Bartonella schoenbuchensis*-IgG antibodies in German forest workers

**DOI:** 10.1186/s13071-026-07526-7

**Published:** 2026-07-28

**Authors:** Kim Nina Buntrock, Wibke Ballhorn, Heike Podlich, Johan Malmström, Lotta Happonen, Sounak Chowdhury, Annette Jurke, Volkhard A. J. Kempf

**Affiliations:** 1 Institute for Medical Microbiology and Infection Control, University Hospital, National Reference Laboratory for Bartonella Infections, Goethe University, Paul-Ehrlich-Str. 40, 60596 Frankfurt Am Main, Germany; 2 Division of Infection Medicine, Lund University, Lund, Sweden; 3 Department Infectious Disease Epidemiology, Northrhine Westphalia Centre for Health, Bochum, Germany; 4 Institute of Hygiene, University Hospital Muenster, University of Muenster, Muenster, Germany

**Correction: Parasites & Vectors (2025) 18:332**

10.1186/s13071-025-06856-2

Following publication of the original article, it came to the author's attention that, owing to a systematic rounding error, the data underlying Fig. 3D were not correct; when using the non-rounded values, the figure was altered slightly. The author's stress that this update to their analysis has no effect on the content of their manuscript and that the following, which they note in their article, remains the case: “Western blot analysis revealed a band reactivity range of 0.1 to 9, again showing no clear differentiation between FW and NFW (Figs. 3, 4) and corroborating the results from IFA".

The figure has since been corrected in the original article. The authors thank you for reading this erratum and apologize for any inconvenience caused.

